# Knowledge, Attitudes and Perception toward COVID-19 Vaccines among Adults in Jazan Province, Saudi Arabia

**DOI:** 10.3390/vaccines9111259

**Published:** 2021-11-01

**Authors:** Edrous Alamer, Faisal Hakami, Sulaiman Hamdi, Afnan Alamer, Mohammed Awaf, Hussam Darraj, Yumna Abutalib, Ebtisam Madkhali, Rahaf Alamer, Nawaf Bakri, Marwa Qadri, Abdullah Algaissi, Abdulaziz Alhazmi

**Affiliations:** 1 Department of Medical Laboratories Technology, College of Applied Medical Sciences, Jazan University, Jazan 45142, Saudi Arabia; ealamer@jazanu.edu.sa (E.A.); AAlgaissi@jazanu.edu.sa (A.A.); 2 Medical Research Center, Emerging and Epidemic Infectious Diseases Research Unit, Jazan University, Jazan 45142, Saudi Arabia; rahafalamer99@gmail.com (R.A.); mqadri@jazanu.edu.sa (M.Q.); 3 College of Medicine, Jazan University, Jazan 45142, Saudi Arabia; faisalhakami1420@gmail.com (F.H.); sulaiman.hamdi27@gmail.com (S.H.); Afnanalameer90@gmail.com (A.A.); hamod6141@gmail.com (M.A.); hosamdarraj25@gmail.com (H.D.); none69991@gmail.com (Y.A.); 1ebtisamahmed@gmail.com (E.M.); nawafbakri@hotmail.com (N.B.); 4 Department of Pharmacology and Toxicology, College of Pharmacy, Jazan University, Jazan 45142, Saudi Arabia; 5 Microbiology and Parasitology Department, College of Medicine, Jazan University, Jazan, 45142, Saudi Arabia

**Keywords:** vaccine, hesitancy, SARS-CoV-2, Saudi Arabia

## Abstract

Background: Saudi Arabia is one of the countries that initiated early vaccination programs despite the global challenges concerning the availability of COVID-19 vaccines. Massive vaccination campaigns have been undertaken in the country; however, negative perception and hesitancy toward vaccines may exist which could reduce public response to vaccination. Further, studies evaluating the current perception and attitude toward COVID-19 vaccines are scarce. Thus, this study aims to assess the community attitudes and perceptions toward COVID-19 vaccines in Jazan Province, Saudi Arabia. Methods: A cross-sectional, retrospective study using an online questionnaire was conducted among the public in Jazan, the southern region of Saudi Arabia. General and demographic data were collected, and perception and attitude toward COVID-19 vaccines were evaluated. Results: Most participants in this study were female (67%) with a median age of 23 years. The majority held a bachelor’s degree, and they trusted the Saudi healthcare system. Our survey showed that 67% of the study participants had positive perceptions toward COVID-19 vaccines, a finding that is significantly associated with receiving the influenza vaccine in the past, the existence of trust on the current healthcare system and holding positive beliefs toward the effectiveness of the current COVID-19 vaccines in reducing the risk of infection, complication, and mortality. Conclusions: The proportion of the public in Jazan who believed in the COVID-19 vaccine effectiveness is not inferior from similar international reports. Thus, national awareness programs toward the effectiveness of the vaccine could be enhanced to accelerate vaccination coverage. Further, nationwide surveys are warranted to include larger populations from different communities to assess the overall perception toward COVID-19 vaccines in the whole country.

## 1. Introduction

Coronavirus Disease 2019 (COVID-19) is an ongoing global pandemic that was declared a global pandemic by the World Health Organization on the 12 March 2020 [1]. This disease is caused by the novel severe acute respiratory syndrome coronavirus 2 (SARS-CoV-2) that belongs to the subgenus Sarbecovirus of the genus Betacoronavirus genera, along with SARS-CoV-1 and a number of emerging animals and bats CoVs [2]. Infection with SARS-CoV-2 can cause a wide variety of clinical manifestations that range from asymptomatic or mild to severe lung and multiorgan diseases which can lead to death [3]. Notably, with the high transmission rates, new variants of SARS-CoV-2 have emerged causing a new challenge in controlling this ongoing pandemic [4]. More than 230 million confirmed cases and ~4.7 million deaths have been reported from all around the world [1]. It is expected that COVID-19 pandemic will continue to impose several burdens of morbidity and mortality, impacting societies and economies worldwide [5]. Vaccination or immunization have been successfully implemented in tackling the global spread of several serious infectious diseases. Immunization can lead to herd immunity which can reduce the circulation of infectious agents among the world population [6]. However, such immunity requires a sufficient proportion of the population to receive the given vaccines [6]. COVID-19 vaccination program has been recognized as an effective measure to ease the burden of the ongoing pandemic; however, the success of this measure can be achieved when there is a high acceptance rate for these vaccines [5,7,8].

Vaccine hesitancy, which is defined as a delay in acceptance or refusal of vaccines despite availability, is reported as a major threat to the effectiveness of vaccination programs [9,10]. The phenomenon of vaccine hesitancy is not novel. Hesitancy towards vaccination was reported since the introduction of the immunization concept by Jenner in the 1800s in Europe against smallpox [1]. Concerns about vaccine hesitancy are growing worldwide [11]. More recently, a report issued by the World Health Organization (WHO) has listed vaccine hesitancy as one of the top ten threats to global health [12]. This conclusion was achieved after a noticeable reduction in the global immunization rates for the measles, mumps, and rubella vaccines, which fell to 85% compared to the required immunization target (95%), resulting in several measles outbreaks around the globe [12]. Anti-vaccination activists against COVID-19 vaccines have mediated the spread of misinformation through multiple channels which may have had a substantial impact on vaccine acceptance [13,14]. Governments and public health sectors must be prepared to address hesitancy and build confidence in vaccination, so immunization would be accepted when implementation is needed. Several factors can affect the public acceptance of pandemic vaccines including, risk perception of the disease, trust in health care systems, past vaccination and general populations’ knowledge about vaccine safety and efficacy, perception of vaccine safety and efficacy, and recommendations from healthcare personnel [15,16,17,18,19,20,21]. Therefore, assessing factors which may mediate hesitancy toward COVID-19 vaccination is essential to reach the required vaccine coverage which will lessen the ongoing pandemic. Therefore, this study aimed to investigate the overall perception towards COVID-19 vaccines in adults residing in Jazan Province, in Saudi Arabia. To accomplish that, an online survey was generated, distributed to our community and the recorded data was statistically analyzed, presented, and discussed.

## 2. Materials and Methods

### 2.1. Study Design and Participants

A cross-sectional, observational study using an online survey to assess the perception of the Jazan public towards COVID-19 vaccines. Jazan is in the southwestern part of Saudi Arabia and has a large population size compared to other provinces of Saudi Arabia. The province harbors almost 2 million inhabitants [22]. Google platform was used to generate a bilingual (Arabic and English) questionnaire which was delivered to participants via social media between 1 July to 24 July 2021. Prior to the distribution of the survey, a pilot sample (*n* = 20) was used to assess the clarity and the wording of the questionnaire items. Of note, data from this pilot sample was excluded from the study analysis. The questionnaire was developed after an intensive search of PubMed for published articles followed by thorough discussions and confirmation by a panel of experts in the field of the study. The study questionnaire was designed to have multiple sections. The survey starts with an introductory part about the purpose of the study, contact details for the study investigators to facilitate communication between study investigators and participants as well as a consent section for agreeing or not to participate in the study. The second section of the study was designed to collect general information and sociodemographic data from participants including age, gender, marital status, presence of chronic diseases, education levels, employment status, type of urbanicity (urban or rural), previous infection of participants or family members with SARS-CoV-2, having concerns of contracting COVID-19, and whether flu vaccines were received in the past. The third section was formulated to focus mainly on the perception and knowledge on the efficacy and the safety of COVID-19 vaccines. This section started with a question to report participants’ trust in the current healthcare system, if sufficient data on vaccine safety and efficacy were provided by the government, and whether the participants believe that it is necessary to apply mandatory vaccination at the workplace. The section also had a subsection to evaluate the awareness of the participants on the safety of the vaccines. This subsection evaluated the overall beliefs of participants on the benefits of the vaccines through questioning participants to report whether they had a positive perception of the vaccines i.e., they believed that the vaccines would provide them with the appropriate immunity required to either reduce the risk of viral infection, to minimize complications of COVID-19 or to decrease the overall mortality rate. Moreover, another subsection was also included in the survey aiming to assess the perception of participants toward the safety of the vaccines. This subsection questioned the participants to report if they believe that COVID-19 vaccines were developed and approved rapidly and if they feel afraid of injections due to potentially serious side effects.

### 2.2. Ethical Approval

Ethical approval for conducting this study was obtained from the ethical approval committee at Jazan University (reference number; REC42/1/087, date 22 March 2021). Consents were taken from all participants prior to participation in the study. The study excluded all participants who refused to participate or those who were outside Jazan Province/Saudi Arabia.

### 2.3. Sample Size and Statistical Analysis

Raosoft sample size calculator (Raosoft Inc., Seattle, WA, USA) was used to calculate the sample size for this study. The population of Jazan Province is estimated to be around 2 million inhabitants [22]. The sample size was calculated based on a 5% margin of error, a 50% response rate, and a 95% confidence interval, for a population of 2 million inhabitants. Consequently, a sample size of 323 responses was determined to be sufficient for this study. However, to reduce sampling bias in our method as this study was based on an online questionnaire distributed via social media, we increased the sample size to include 636 participants which is more than 2-folds of the required size. Descriptive statistics were reported for the collected data. t-test and chi-square test were both performed for statistical analysis. The data were statistically analyzed using SPSS v.23 (IBM Corp., Armonk, NY, USA) with the α criterion for the *p*-value set at 0.05.

## 3. Results

### 3.1. Sociodemographic Characteristics of the Study Subjects

A total of 655 participants completed the survey and were included in the study analysis. The median age for the study participants was 23 years old, with about 33% males and 61% females. These data are also summarized in Table 1. More than two-thirds of the study participants were singles (66%), almost one-third of the respondents were married (31%), and 1% and 2% were widows or divorced, respectively. Study respondents showed various educational backgrounds with most of them having undergraduate degrees (69%), followed by 30% of participants holding high school degrees and only 1% of the participants reported to have postgraduate degrees. Most of the study participants were students (60%), 26% of the participants had occupations (governmental or private sectors), and only 2% and 12% of the study participants were retired or unemployed at the time of conducting this study, respectively. Most of the study subjects were from rural areas (68%). Chronic diseases were reported by only 13% of the study participants. 

A large number of the participants trusted the Saudi health care system (91%) with a few percent of the participants reported receiving the influenza vaccines in the past (33%). Worry about COVID-19 was reported by 69% of the study subjects and previous SARS-CoV2 infections were reported by 22% and 35% of the participants or their close family members, respectively. Most of the study participants ranked the risk of COVID-19 as moderate (57%) and almost 34% of the participants classified COVID-19 as a high-risk disease with only a few participants (9%) believed that the risk is low.

### 3.2. Knowledge, Attitude, Practices, and Health Beliefs on COVID-19 Vaccines

In the present study, 428 of the study participants (representing 67% of the total study subjects) believed that COVID-19 vaccines would provide immunity against COVID-19 (Table 2). 88% of the participants thought that current vaccines will help in reducing the potential risk of SARS-CoV-2 infection, and 94% and 88% of them believed that these vaccines would minimize COVID-19 complications and mortality, respectively. Almost 85% of the participants were satisfied and felt that they had received sufficient information about vaccines’ safety and efficacy and therefore, 86% of the participants thought that the vaccines should be mandatory at the workplace. 88% of the participants believed that these vaccines are recommended by trusted healthcare professionals. Almost 95% of the study participants received the vaccines, and 92% of the subjects will recommend their family members to take COVID-19 vaccines. More importantly, 81% of the participants will continue to practice preventive measures after receiving COVID-19 vaccines (Table 2). Based on participants’ perception of COVID-19 vaccine efficacy, we categorized our study participants into two main groups, i.e., participants with a negative perception on vaccine efficacy who did not believe that the vaccines produce immunity against COVID-19 and those who had a positive perception of vaccine efficacy (Table 3). These two groups were then compared against each other using several parameters including age, gender, marital status, education levels, employment status, confidence in the current health system, the existence of chronic diseases, receiving influenza vaccines in the past, and prior history of participants or family members with SARS-CoV2. Moreover, more parameters were also compared between these two groups including overall knowledge on vaccines i.e., receiving sufficient information on vaccine efficacy, the necessity to apply mandatory vaccination at workplaces, the efficacy of the current vaccines in reducing infection, disease complication, and mortality. Additionally, we also tested other parameters which may mediate the negative perception on COVID-19 vaccines including questioning whether participants believe that the vaccines were rapidly developed and produced and if they were concerned about some potentially serious side effects following vaccination. As shown in Table 3, the Chi-square test and t-test for univariate showed that several factors have significant associations with negative perception on COVID-19 vaccines. These include the levels of trust on health care system (*p* = 0.002), receiving influenza vaccine in the past (*p* = 0.001), and the level of knowledge on COVID-19 vaccines, i.e., the availability of sufficient information about the vaccines (*p* = 0.0001), effectiveness of the vaccines to reduce either risk of infection (*p* = 0.0001), disease complication (*p* = 0.0001) or mortality (*p* = 0.0001). Significantly more participants with positive perception of vaccine believed that the vaccines are recommended by trusted healthcare professionals (*p* = 0.0001) and should be mandatory at the workplace (*p* = 0.0001). More participants with negative perception believed that the vaccines may induce serious side effects (91%) compared to those in the positive perception group (84%). More participants in the negative perception thought that the vaccines were developed and produced rapidly (82%) compared to participants in the positive perception group (78%); however, this was not significantly different. We further analyzed the impact of other factors on vaccine perception including the existence of chronic diseases, previous history of participants or family members with SARS-CoV-2 infections, and the overall concerns about COVID-19. Our data analysis showed that none of these factors had significant associations with positive or negative perception towards vaccines (Table 3).

## 4. Discussion

Vaccination is an effective way to lessen the burden of the ongoing global pandemic of COVID-19. The current key goal of most countries is to vaccinate rapidly to reach the proper herd immunity which is required to stop the spread of this ongoing pandemic. The urgency of the pandemic resulted in the rapid development of COVID-19 vaccines which may have boosted the hesitancy towards receiving the vaccines among some individuals. This hesitance is associated with vaccine efficacy and safety [5]. In general, hesitancy toward vaccines is a frequently reported concern whenever a new vaccine is introduced to the population [21,23]. Therefore, identifying factors that may mediate population hesitancy on COVID-19 vaccines must be identified and addressed which may increase population confidence and acceptability of COVID-19 vaccines. In contribution to this, we have conducted a cross-sectional, observational study to assess the overall perception of adults on the efficacy of the currently approved COVID-19 vaccines in Jazan province, Saudi Arabia.

In this web-based study, we collected data from adults residing in the province of Jazan, southern region of Saudi Arabia, and analyzed participants attitudes and perceptions towards COVID-19 vaccines which were recently approved in the country. In Saudi Arabia, three vaccines have been approved yet including Pfizer-BioNTech, Oxford-AstraZeneca, and more recently, Moderna mRNA-1273 COVID-19 vaccines [24]. These vaccines have shown high efficacy and safety profiles in preclinical and clinical studies. Phase III trials showed that BNT162b2 has about 95% efficacy against laboratory-confirmed SARS-CoV-2 symptomatic infection, at least seven days after the second dose in the individual of 16 years and older without current or previous history of COVID-19 [25,26]. Two doses of Oxford-AstraZeneca adenovirus-vectored vaccine (ChAdOx1nCoV-19) showed an overall 62.1% efficacy against symptomatic SARS-CoV-2 infection [27]. Data from the phase III clinical trial also showed that Moderna mRNA-1273 showed 94.1% efficacy for the prevention of symptomatic SARS-CoV-2 infection as compared with placebo [28]. A previously reported study showed that more participants ranked the efficacy of COVID-19 vaccine as moderate or low [29]. However, data from the current study showed that 67% of the study participants had positive perceptions on the currently available COVID-19 vaccines given in Saudi Arabia. Our data is consistent with a recent worldwide systematic review about vaccination willingness in which they found that about 66% have a positive attitude toward COVID-19 vaccines [30]. Variation in vaccine perception in the above studies could be due to the difference in the timing of conducting these studies as more confidence was built following the global mass vaccination campaigns which were conducted in the last few months. Similar trends were also observed during the influenza pandemic where intense worries and concerns on vaccines were reported during the early days of the pandemic; however, these worries lessened as the pandemic progressed, reporting subsequent increase in acceptance rate for vaccines by the general population [31,32]. Previous studies on vaccine acceptance demonstrated that many factors could affect the acceptance of pandemic vaccines including trust in health care systems, past vaccination, perception on vaccine safety and efficacy, and vaccine recommendations from doctors [15,16,17,18,19,20,21]. The present study showed that previous history of vaccination against influenza and general knowledge about COVID-19 were significantly associated with positive perception toward COVID-19 vaccines among our study participants (Table 3). More respondents in the negative perception group reported no trust in the current healthcare system (15%) compared to those with positive perception (7%). 94% of the participants in the positive perception group believed that COVID-19 vaccines were recommended by trusted doctors/healthcare professionals compared to only 78% in the negative perception group. Consequently, 94% in the positive perception group thought that COVID-19 vaccines should be mandatory in the workplace. Previous studies [5,33,34] showed that trust is a positive indicator for vaccine acceptance. Moreover, it was shown that the acceptance of the COVID-19 vaccines decreased as the hypothetical effectiveness of the vaccine decreased [35]. Furthermore, lack of trust in health authorities was one of the most frequently reported barriers to vaccine uptake in general [36]. A more recent and global study also showed that a high percentage of vaccine acceptance was reported among individuals who reported trust in their government compared to those with less trust [5]. This study also showed that individuals who reported higher levels of trust in information from government sources including healthcare sectors were more likely to accept the vaccines [5]. These data highlight the importance of enhancing the trust between healthcare sectors and populations which can be achieved by increasing transparency and making more efforts to approach the community. The seasonal flu vaccine is a well-known and largely used vaccine among different age groups among world population. Here in our study, we also used this factor to assess the impact of previous vaccination experience on participants’ perception towards COVID-19 vaccines. Our data showed that a history of vaccination with flu vaccines was frequently reported by the positive perception group compared to those in the negative perception group, at 71% and 59%, respectively. Several other studies also reported positive trends in the acceptance rate of COVID-19 vaccines among individuals with a previous history of influenza vaccines [8,37,38,39]. In a study conducted on healthcare workers aiming to assess the association of health behavior with influenza vaccination, it was found that those who did not receive flu vaccines believed that preventive measures such as handwashing were more effective than vaccination [40]. Data from our study and all the above-mentioned studies highlight the positive role of vaccination history in establishing constructive beliefs on vaccination which are consistent with several previous studies [15,41]. Therefore, extending the education programs for regular vaccination including flu and other vaccines for non-pandemic infectious diseases may contribute to improving the overall confidence of the public toward vaccination which is required for potential future pandemics. Furthermore, lack of information on the current COVID-19 vaccines was shown to be a major driver of vaccine hesitancy [42]. A recent study on healthcare workers showed that lack of knowledge and evidence on COVID-19 vaccines mediated the unwillingness to receive COVID-19 vaccines [43]. Further analysis on vaccine knowledge of our study data showed that respondents in the positive perception group received sufficient information on vaccine safety and efficacy compared to the negative perception group (*p* = 0.0001), at 93% and 69%, respectively. Respondents in the negative perception group reported significantly lower perception on vaccine ability to reduce the risk of infection (72%,), reduce COVID-19 complication (83%), reduce mortality (73%), compared to their counterparts, at 95%, 99%, 95%, and 78%, respectively. More importantly, concerns on potential side effects were more frequently reported by individuals with negative perceptions (*p* = 0.015). Although robust information on vaccines safety and efficacy are available and circulating in the media, negative information still exists which can be too overwhelming for the general population to comprehend. A recent study showed that among individuals with intermediate hesitance toward COVID-19 vaccines, the intention to receive the vaccines increased after providing the right information about the safety and efficacy of COVID-19 vaccines compared to those who did not receive the same knowledge [42]. Therefore, findings from our study and others emphasize the need of enhancing the delivery of science including the safety and efficacy of vaccines to the public. Here, continuous intervention by experts to convey correct knowledge about vaccines is warranted.

To our knowledge, this study is one of very few qualitative studies which assessed factors influencing overall perception toward COVID-19 vaccines in Saudi Arabia. This study identified several factors which mediated the negative perception toward the COVID-19 vaccines including trust in the healthcare system, previous history of influenza vaccine, and the overall knowledge of participants on COVID-19 vaccines. Data from this study may be useful in designing effective immunization education programs for the general population which is needed to increase the acceptance rate for COVID-19 vaccines as well as for the potential future endemics and pandemics. Nevertheless, this study has several limitations. As this study was conducted during COVID-19 pandemic, a self-reported survey was used which may introduce information bias. Another limitation of the current study is the usage of small sample size. Therefore, future studies with larger sample size are warranted. Additionally, the distribution of this survey was dependent on the authors’ networks. It would be more appropriate to conduct a community-based or national survey for this kind of study. Another limitation of this study is that we have chosen limited and specific factors which we believe to have the most influencing effects on perception towards the vaccines. Therefore, future studies with extensive factors are warranted. It is noteworthy that the total number of respondents in the positive perception arm is more than 2-fold than those in the negative perception group. However, the negative perception group showed significantly higher percentage (91%) compared to the positive perception group (84%) (Table 3), and both numbers considered high. In the recent months, some rare side effects including clotting and myocarditis were reported [44], and these may still drive some concerns among the majority of our study participants including individuals in the negative perception and positive perception groups. These findings cannot be directly justified, and further national studies are required for better understanding the major drivers toward vaccines positive and negative perceptions.

## 5. Conclusions

In general, most participants in our study trusted the healthcare system in Saudi Arabia and they positively believed in the effectiveness of available COVID-19 vaccines. These positive beliefs are significantly correlated with adequate knowledge about the effectiveness of the COVID-19 vaccine in minimizing the SARS-CoV-2 risk of transmission, complications, and mortality. Our results are generally consistent with many reports about COVID-19 vaccines perception and attitude around the globe. Thus, we believe that national efforts to increase the awareness toward COVID-19 vaccines should be continued to reach a satisfied coverage of vaccination that can assure reaching the targeted herd immunity. Consequently, national surveys on a larger population and various communities in a country with about 35 million inhabitants should be considered to have a better understanding of the current perception toward COVID-19 vaccines.

## Figures and Tables

**Table 1:** General characteristics of the study participants.

Variable	Participants, *n* = 655
Age, years (Median; SD)	23;9
Male, *n* (%)	217 (33%)
Marital status, *n* (%)	
Single	436 (66%)
Married	203 (31%)
Widow	4 (1%)
Divorced	12 (2%)
Highest education level, *n* (%)	
High school	193 (30%)
Bachelor’s degree	452 (69%)
Postgraduate level	10 (1%)
Occupation, *n* (%)	
Governmental	139 (21%)
Private	36 (5%)
Student	388 (60%)
Retired	12 (2%)
unemployed	80 (12%)
Living, *n* (%)	
City	211 (32%)
Village	444 (68%)
Chronic disease, *n* (%)	
Yes	83 (13%)
No	572 (87%)
Trust in the health care system, *n* (%)	
Yes	593 (91%)
No	62 (9%)
Received influenza vaccine, *n* (%)	
Yes	215 (33%)
No	440 (67%)
Contracted COVID-19, *n* (%)	
Yes	147 (22%)
No	508 (78%)
One of your family contacted COVID-19, *n* (%)	
Yes	428 (65%)
No	227 (35%)
Worried about COVID-19, *n* (%)	
Yes	453 (69%)
No	202 (31%)
The risk of COVID-19 to people in the Jazan region, *n* (%)	
Low	59 (9%)
Moderate	374 (57%)
High	222 (34%)

SD: Standard deviation.

**Table 2:** Knowledge and practice of the study participants toward COVID-19 Vaccine.

Question	Participants, *n* = 655
Positive perception on vaccines: Believe that the vaccines would produce immunity against COVID-19, *n* (%)	
Yes	428 (67%)
No	227 (33%)
Vaccination helps to reduce the risk of virus infection, *n* (%)	
Yes	574 (88%)
No	81 (12%)
Vaccination will reduce complications of COVID-19, *n* (%)	
Yes	615 (94%)
No	40 (6%)
Vaccine is effective to reduce mortality from COVID-19, *n* (%)	
Yes	575 (88%)
No	80 (12%)
There is sufficient information regarding the vaccine’s safety and efficacy released by the government, *n* (%)	
Yes	559 (85%)
No	96 (15%)
Vaccine is recommended by a trusted doctor/healthcare professional, *n* (%)	
Yes	579 (88%)
No	76 (12%)
Vaccine should be mandatory at the workplace, *n* (%)	
Yes	565 (86%)
No	90 (14%)
Received COVID-19 vaccine, *n* (%)	
No	30 (5%)
Yes	625 (95%)
First Dose	213 (32%)
Second Dose	411 (63%)
I’ll recommend my family to take the COVID-19 vaccine, *n* (%)	
Yes	605 (92%)
No	50 (8%)
No need to do the preventive measures after receiving COVID-19, *n* (%)	
Yes	124 (19%)
No	530 (81%)

SD: Standard deviation.

**Table 3:** Information about Participants Perception on COVID-19 vaccines.

Variable	Negative Perception on Vaccines: Aren’t Confident about COVID-19 Vaccine, *n* = 217 (33%)	Positive Perception on Vaccines: Are Confident about COVID-19 Vaccine, *n* = 438 (67%)	*p*-Value #
Age, years (mean; SD)	26.8	27.9	0.492
Gender			
Male, *n* (%)	46 (21%)	171 (39%)	0.0001 *
Female	171 (79%	267 (61%)	
Marital status, *n* (%)			
Single	164 (76%)	252 (58%)	0.005 *
Married	50 (23%)	153 (31%)	
Widow	0 (0%)	4 (1%)	
Divorced	3 (1%)	9 (2%)	
Highest education level, *n* (%)			
High school	54 (25%)	139 (32%)	0.185
Bachelor degree	159 (73%)	293 (67%)	
Postgraduate level	4 (2%)	6 (1%)	
Occupation, *n* (%)			
Governmental	43 (20%)	96 (22%)	0.872
Private	11 (5%)	25 (6%)	
Student	136 (63%)	252 58%)	
Retired	3 (1%)	9 (2%)	
unemployed	24 (11%)	56 13%)	
Chronic disease, *n* (%)			
Yes	20 (9%)	63 (14%)	0.062
No	197 (91%)	375 (86%)	
Trust in health care system, *n* (%)			
Yes	185 (85%)	408 (93%)	0.002 *
No	32 (15%)	30 (7%)	
Vaccine is recommended by a trusted doctor/healthcare professional, *n* (%)			
Yes	169 (78%)	410 (94%)	0.0001 *
No	48 (22%)	28 (6%)	
Vaccine should be mandatory at the workplace, *n* (%)			
Yes	155 (71%)	410 (94%)	
No	62 (29%)	28 (6%)	0.0001 *
Received influenza vaccine, *n* (%)			
Yes	127 (59%)	313 (71%)	0.001 *
No	90 (41%)	125 (29%)	
Contracted COVID-19, *n* (%)			
Yes	161 (74%)	347 (79%)	0.164
No	56 (26%)	91 (21%)	
One of your family contacted COVID-19, *n* (%)			
Yes	152 (70%)	276 (63%)	0.081
No	65 (30%)	162 (37%)	
There is sufficient information regarding the vaccine’s safety and efficacy released by the government, *n* (%)			
Yes	150 (69%)	409 (93%)	0.0001*
No	67 (31%)	29 (7%)	
Vaccination helps to reduce the risk of virus infection, *n* (%)			
Yes	156 (72%)	418 (95%)	0.0001 *
No	61 (28%)	20 (5%)	
Vaccination will reduce complications of COVID-19, *n* (%)			
Yes	181(83%)	434 (99%)	0.0001 *
No	36 (17%)	4 (1%)	
Vaccine is effective to reduce mortality from COVID-19, *n* (%)			
Yes	158 (73%)	417 (95%)	0.0001 *
No	59 (27%)	21 (5%)	
COVID-19 vaccine was rapidly developed and approved, *n* (%)			
Yes	179 (82%)	341 (78%)	0.183
No	38 (18%)	97 (22%)	
There are some possible serious side effects of vaccination, *n* (%)			
Yes	198 (91%)	369 (84%)	0.015 *
No	19 (9%)	69 (16%)	

SD: Standard deviation; # The α criterion for *p*-value was set to 0.05; * Significant in univariate analysis.

## Data Availability

The data presented in this study are available on request from the corresponding author.
